# Supplementary figures and images for: Timing urinary tract reconstruction in rats to avoid hydronephrosis and fibrosis in the transplanted fetal metanephros as assessed using imaging
Source: PLoS One. 2021 Jan 15;16(1):e0231233. doi: 10.1371/journal.pone.0231233 (PMC7810319; doi:10.1371/journal.pone.0231233)

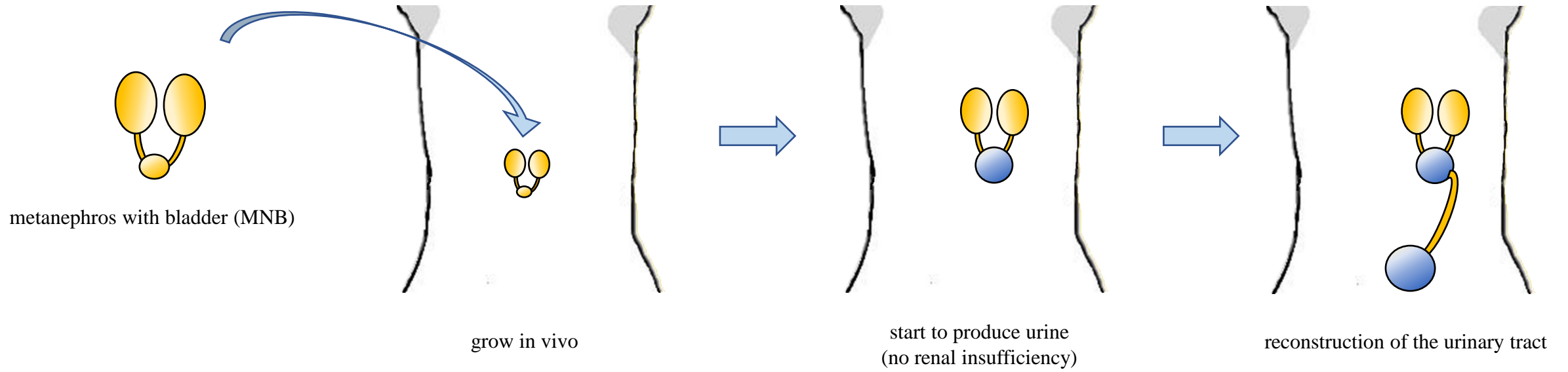

Supplement: S1 Fig — The metanephros with bladder (MNB) is transplanted in the recipient and allowed to grow. The ureter of the recipient is anastomosed to the MNB bladder where urine has accumulated. In this manner, urinary excretion from the MNB can be measured. (PDF) [file pone.0231233.s001.pdf]
