# Supplementary material for: Timing urinary tract reconstruction in rats to avoid hydronephrosis and fibrosis in the transplanted fetal metanephros as assessed using imaging
Source: PLoS One. 2021 Jan 15;16(1):e0231233. doi: 10.1371/journal.pone.0231233 (PMC7810319; doi:10.1371/journal.pone.0231233)
Supplement: S1 Table — Measurements are performed under the same conditions as those maintained for the GFR measurement method in the metanephros with the bladder. (PDF) [file pone.0231233.s002.pdf]

Rat number	GFR (mi/min/m^2)
1	58.7
2	58.0
3	64.4
4	65.2
Average	61.6
Standard Deviation	3.2
