# Supplementary material for: Timing urinary tract reconstruction in rats to avoid hydronephrosis and fibrosis in the transplanted fetal metanephros as assessed using imaging
Source: PLoS One. 2021 Jan 15;16(1):e0231233. doi: 10.1371/journal.pone.0231233 (PMC7810319; doi:10.1371/journal.pone.0231233)
Supplement: Attachment — Submitted filename: Response_to_Reviewers.docx [file pone.0231233.s003.docx]

August 15, 2020

Dr. Franziska Theilig

Academic Editor

*PLOS ONE*

Dear Editor:

I am terribly sorry for the late reply. Thank you for inviting us to submit a revised draft of our manuscript newly entitled, “**Timing urinary tract reconstruction in rats to avoid hydronephrosis and fibrosis in the transplanted fetal metanephros as assessed by imaging**.” The original submission to *PLOS ONE* was on Mar 17th, 2020 and the manuscript ID number is PONE-D-20-07683.

We appreciate the time and effort you and each of the reviewers have dedicated to provide insightful feedback on ways to strengthen our paper. Thus, it is with great pleasure that we resubmit our article for further consideration with the incorporated changes that reflect the detailed suggestions you have graciously provided. We hope that our edits, highlighted in the manuscript, and the response to reviewers provided below, satisfactorily address all of the issues noted.

Thank you for your consideration. I look forward to hearing from you.

Sincerely,

Satomi Iwai

Associate professor

Laboratory of Small Animal Surgery 2

School of Veterinary Medicine

Kitasato University

35-1, Higashi23, Towada-city, Aomori, 034-8628

E-mail: [iwai@vmas.kitasato-u.ac.jp](mailto:iwai@vmas.kitasato-u.ac.jp)

**RESPONSE TO REVIEWERS**

***Response to Reviewer 1:*** I am very grateful for the valuable time and effort that you have put into reviewing my paper. I am aware that corrections were necessary throughout the manuscript, and I have revised the text in accordance with your suggestions. In addition, you indicated that the statistical analysis was not appropriate. In response, I have rechecked the statistical methods. Please let me know if there is a need for further corrections.

**Major comments**

1. The whole manuscript should be extensively proofread by a native English speaker. In the present form, it is difficult to evaluate the correctness of the results or the logical flow of the discussions. Thus, I have not commented on the Discussion section in this round of review.

**Response:** Thank you for your comment. I have resubmitted the manuscript for editing by a native English speaker. Proof of the editing service is attached.

2. The experimental designs (Experiments 1, 2, and 3) and sample definitions (such as MNB 2, SP, and 28UR) should be included in the Results section, so that readers can understand the aims of the experiments.

**Response:** Thank you for your comments. In response to your concerns, I have included the sample definitions in the Results section. ***(Lines 292-298, 375-380, 408-410)***

3. The descriptions in the Methods section should be limited to detailed technical issues.

**Response:** I have added an experiment flow chart to the Materials and methods section as a new figure to clarify the aims of the experiments.

***(Line 134, 197, 224):*** The flow of the experiment is shown in Fig 1A, B, C.

4. The present Methods section lacks some important points, such as how the areas of tubule dilatation or fibrosis were calculated per slide and how many slides were analyzed per kidney.

**Response:** Thank you for your careful review. I have added text in the Methods section regarding the evaluation of the metanephros slides, which describes in detail the methods used.

***(Lines 256-262):*** “One slide was randomly selected to acquire images and all tests were blindly observed from multiple slides by three people. HE, MT, TGF-β1, collagen-α1 type 1, and vimentin measures were based on 20 non-overlapping images and calculated based on the average area. For an evaluation by TUNEL staining, apoptosis of the tubular and glomerular cells was counted in five non-overlapping images observed at 400× magnification and the ratio of these cells to unstained cells was calculated.”

5. The number of Figures should be reduced. Furthermore, the experimental designs, which are currently presented as Supplementary Figures, can be incorporated into the main Figures. For example, the design for Experiment 1, Fig. 1, and Fig. 3 can be combined into a single Figure. Likewise, the design for Experiment 2, Fig. 4, and Fig. 5 can be combined, as can the design for Experiment 3, Fig. 6, and Fig. 7. Figs. 8–10 can also be combined.

**Response:** Thank you for your suggestion. There are now a total of 5 figures and one supplementary figure. Specifically, Figures 1 to 3 are now combined into Figure 2. Figures 4 and 5 are now combined into Figure 3. Figures 7 to 10 have been combined into Figure 5.

6. The TUNEL staining in Fig. 10 is too weak and hardly different from the background signals. Replace with more convincing photos or delete the whole figure.

**Response:** Thank you for your comment. I tried to stain again, but the original sample was too small to be used for other analyses. As a result, reference to this stained image was deleted from the manuscript.

7. It is not clear how many samples were processed in each experiment. Clearly indicate the sample numbers in each Figure and Table or their corresponding legends.

**Response:** Thank you for your suggestion. An experiment flow chart with the number of samples has been inserted as a new figure from the supplemental data.

***(Line 134, 197, 224):*** The flow of the experiment is shown in Fig 1A, B, C.

8. Explain each figure panel one by one in the text. In the present form, the contribution of each panel to the logical flow of the text remains unclear.

**Response:** Thank you for your suggestion. We improved the logical flow to present the figures and added more detail to the description of each panel. Each panel has been referenced in the manuscript, indicated by a corresponding figure highlighted in the text*.*

9. In the Abstract, the authors claimed that they generated iPS cell-derived kidneys, but this is not supported by the literature. In the Introduction section, the authors claim that they generated iPS cell-derived kidney tissues, but the cited references only report data using mouse, rat, or mesenchymal stem cells. If this is done intentionally, it is a serious violation against scientific rules and ethics. The authors should avoid presenting a misleading Introduction section by correctly explaining their own achievements and fairly citing the key papers on iPS cell-derived organoids. At present, only two references are cited (references #3 and #4), and they are misinterpreted.

**Response:** Thank you for your comments. We have clarified the information presented in the Abstract and Introduction and re-evaluated our understanding of the information presented in references #3 and #4.

***(Lines 48-49): “***Thus, this study contributes to the evidence base for the possibility of kidney regeneration in human clinical trials.”

***(Lines 61-63)*** “For example, there has been an attempt to develop a kidney using pluripotent stem cells. Some studies have experimented with a method to produce kidney or nephron and ureteric bud progenitor cells *in vitro* by using human stem cells [3,4].”

10. The title does not convey the precise message. This manuscript deals with transplanted kidneys. Consider revising the title.

**Response:** Thank you for your careful reading. We have changed the title to, “**Timing urinary tract reconstruction in rats to avoid hydronephrosis and fibrosis in the transplanted fetal metanephros as assessed by imaging**.”

**Minor comments**

1. Fig. 2 is actually a Table. Therefore, present it as a Table. Alternatively, it can be incorporated into other Figures.

**Response:** Thank you for your suggestion. Figure 2E is now shown in Table 2.

2. Do Figs. 7A and 8A represent the data before 3 weeks or at 8 weeks after transplantation? The Figure Legends suggest the former, but the actual Figures appear to indicate the latter.

**Response:** Thank you for your question. Figures 7 and 8A are now a part of Figure 5, which shows MNBs removed 8 weeks after transplantation. As you pointed out, the figure legends were difficult to understand and they have been revised for clarity. ***(Lines 445-449)***

3. Table 1 is not cited in the text.

**Response:** Thank you for your comment. Table 1 is now referred to in the Results section.

***(Lines 304-307):*** “The ultrasonographic examination allowed for early stage detection of the MNB that was present near the aorta, with clear margins. The ultrasonographic examination also allowed for 100% recognition of both the MNB1 (the first transplanted MNB at Week 0) and MNB2 (the second transplanted MNB at Week 4) at 3 weeks after transplantation (Table 1), and partial observation of the newly formed blood vessels around the MNB was made using the color Doppler method (Fig 2D).”

Figs. S1 and S2 are mislabeled.

**Response:** Thank you for your careful reading of our manuscript. There is now only one supplementary figure, which is labelled “S1 Fig” ***(Line 81)***.

4. The Tajiri Scientific Reports paper remains uncited on page 5.

**Response:** Thank you for your comment. I rechecked the references and the Tajiri Scientific Report paper is reference #10, cited in the text on ***Line 76***.

5. The explanation of Fig. 5 in the text appears to be opposite to the actual data.

**Response:** Thank you for your comment. Figures 5 is now a part of Figure 3 B, C. I rechecked the sentence and revised it. “The degree of tubular dilation in the removed MNBs before than Day 21 was significantly different compared to that after 21 days from transplantation *(Line 400-403).* There is no significant difference in fibrosis.

6. An incorrect paper (reference #2) is cited on page 26.

**Response:** Thank you for your comment. I rechecked and corrected the references. The proper reference number is #11, cited first in the text on ***Line 80.***

***Response to Reviewer 2:*** Thank you very much for your valuable time spent reviewing my manuscript.

1. Is it able to apply for clinical application although human body size is larger than that of rats?

**Response:** Thank you for your question. In the present study, we transplanted the MNB near the abdominal aorta by ultrasonography and used it as a reference. In humans, the abdominal aorta can also be confirmed ultrasonography. In addition, other animal species larger than rats can be found for experiments because the MNB to be transplanted also becomes larger. Since we hope to continue to use larger animals, knowledge regarding the timing for reconstruction will be increasingly useful.

2. What do you think about the function other than the production of urine, for instance, renin?

**Response:** Thank you for your question. We have also considered further functional evaluation of the MNB. I believe that the results of this study will greatly affect the physiological functions such as the measurement of renin-angiotensin system (RAS) and the amount of erythropoietin (EPO) production. It has been pointed out that the RAS system is related to EPO production, and it is known that EPO decreases early with the onset of hydronephrosis (2013, Souma T, J Am Soc Nephrol). In addition, EPO is known to improve the function of obstructive kidney damage by anti-inflammatory and anti-apoptotic effects (2020, Park E, Cell Stress and Chaperones) and is considered to be deeply involved in maturation of the metanephros.

Based on your question, we have added more comments to the limitations section of our study.

***(Lines 614-616):*** First, we only performed diagnostic imaging-based assessments and did not assess other physiological aspects such as renin and erythropoietin activity.

***Response to Reviewer 3:*** Thank you for taking the time to comment on our manuscript. I apologize for the mistakes that made it difficult to read the text. I have made corrections in consideration of your comments and have worked hard to incorporate your feedback.

**Major points:**

1. There seems to be many grammatical and typographical errors and inaccurate descriptions, which makes this manuscript difficult to understand. The English should be revised throughout the manuscript by a native English speaker.

**Response:** Thank you for your advice. I have resubmitted the manuscript for editing by a native English speaker. I believe that this has greatly improved the quality of the language.

2. One of the two criterion for the appropriate timing of urinary tract reconstruction used in this study is the MNB bladder volume larger than 0.016 cm3. This reviewer is wondering how they decided the specific volume (0.016 cm3). The data comparing 0.016 cm3 and other volumes or the explanation of the process to make the criterion should be added.

**Response:** Thank you for your comment. The present study showed that the bladder volume of the MNB was highly correlated with findings from CT and ultrasonography and that the actual volume of urine is highly correlated with the volume calculated by ultrasonography. The three observers randomly performed the ultrasonic examinations and calculated the results to provide stable and accurate values regardless of differences in skill. Furthermore, the person performing the urinary tract reconstruction has routinely used microsurgery techniques, and previous studies have reported similarly performed anastomosis in MNBs (2015, Yokote S, PNAS). Previous reports have shown that the tubules of the anastomosed MNB have little dilation and the histopathological examination and contrast-enhanced CT examination have confirmed patency of the anastomotic site. Therefore, in this study, as well as in the previous study, using the size of the MNB bladder, which showed less dilatation of the tubules after anastomosis, as the regulation of the optimal anastomosis period made it possible to reconstruct the urinary tract as early as possible. I added this to the section ~ ***lines 144-148, 196-215*** in the text.

3. The images of Figure 10A are unclear. It is now difficult to find the TUNEL (+) apoptotic cells and glomeruli. The clearer images are required.

**Response:** Thank you for your comment. I received similar comments from the other reviewers and since I was unable to get a better-quality image, this figure has been deleted from the manuscript. Figures 10 are now a part of Figure 5,

Minor points:

1) Abstract, line 24. “iPS-derived generated kidney” should be replaced by “iPS cell-derived regenerated kidney”.

**Response:** Thank you for your comment. This term has been revised in response to comments from other reviewers. I have deleted the words related to incorrect referencing.

2) Abstract, line 37. “transforming growth factor-β” should be replaced by “transforming growth factor-β1.”

**Response:** Thank you for your keen observation. This has been revised ***(Line 45).***

3) Abstract, line 40. “kidney generation” should be replaced by “kidney regeneration.”

**Response:** Thank you. I have made the revision ***(Line 49).***

4) Introduction, line 50. “a need treatment for alternatives” is inaccurate. Should the description be “a need for alternative treatment”?

**Response:** Thank you for your suggestion. I have made the revision ***(Line 59).***

5) Introduction, line 52. “Takasato et al., [3,4]” is wrong. The ref. [4] was published by another group.

**Response:** Thank you for your keen observation. I have rechecked and revised the references. Please refer to ***Lines 62-64.***

6) Introduction, line 71. “this success” should be replaced by “these successes”.

**Response:** Thank you for your comment. This has been revised in ***Line 77.***

7) Introduction, line 76. SWPU is shown now in “S2 Fig” but not in “S1 Fig”.

**Response:** I apologize for the mistake in the annotation. I have made many changes to the figures in response to the other reviewers’ comments. Along with other changes, the number was corrected.

8) Materials and methods, line 234. “20 taken” should be replaced by “20 images taken”.

**Response**: Thank you. This has been revised in ***Line 259***.

9) Results, lines 303 and 304. Is this data “data not shown”?

**Response:** Thank you for your question. I have added a graph showing the correlation between actual MNB urine volume and ultrasonic urine volume (Fig. 2F) ***(referenced in Line 361-364).***

10) Results, lines 314-320. Are these data “data not shown”?

**Response:** Thank you for your question. It has already been reported that the anastomosis compared with non-anastomosis has a mild urinary dilatation due to the urination route, and the same results have been described (Reference 12). To clarify, I added "data not shown" in ***Line 369.***

11) Results, lines 341-343. Is this sentence accurate? Figure 4A seems to indicate that the MNBs removed 21 or more days after transplantation had significantly more severe tubular dilation than those removed less than 21 days after transplantation.

**Response:** Thank you for bringing this to my attention. The text was incorrect. I made a new figure and revised the text.

***(Lines 396-403):*** Comparison of tubular dilation in the MNBs removed before and after 21 days from transplantation. The degree of tubular dilation in the MNBs before day 21 was significantly different compared to that after 21 days from transplantation (p < 0.01. C) Comparison of interstitial fibrosis in the MNB removed before Day 21 from transplantation and after 21 days from transplantation.

12) Results, line 378. “tubular” should be replaced by “tubules”.

**Response:** Thank you for your suggestion. This word has been changed due to the integration of figures. It currently exists in the legend in Figure 5B ***(Line 447-448).***

13) Results, lines 396 and 398. “interstitial” should be replaced by “interstitium”.

**Response:** Thank you for your suggestion. Revisions were made in ***Line 430.***

14) Figure 10 legends do not correspond to the figures. The legend B) explains lower panels in Figure A, and the legend describing Figure B is missing.

**Response:** Thank you for pointing this out. Many of the figures in the manuscript have been combined and revised for resubmission.
