# Supplementary material for: Timing urinary tract reconstruction in rats to avoid hydronephrosis and fibrosis in the transplanted fetal metanephros as assessed using imaging
Source: PLoS One. 2021 Jan 15;16(1):e0231233. doi: 10.1371/journal.pone.0231233 (PMC7810319; doi:10.1371/journal.pone.0231233)
Supplement: Attachment — Submitted filename: Response_to_Reviewers.docx [file pone.0231233.s004.docx]

November 5, 2020

Dr. Franziska Theilig

Academic Editor

*PLOS ONE*

Dear Editor:

Thank you again for inviting us to submit a revised draft of our manuscript entitled, “**Timing urinary tract reconstruction in rats to avoid hydronephrosis and fibrosis in the transplanted fetal metanephros as assessed using imaging**.” The original submission to *PLOS ONE* was on Mar 17th, 2020. The manuscript ID number is PONE-D-20-07683.

We appreciate the time and effort you and each of the reviewers have dedicated to provide insightful feedback on ways to strengthen our paper. Thus, it is with great pleasure that we resubmit our article for further consideration with the incorporated changes that reflect the detailed suggestions you have graciously provided. We hope that our edits, highlighted in the manuscript in red font, and the response to reviewers provided below have satisfactorily addressed all of the issues noted.

Thank you for your consideration. I look forward to hearing from you.

Sincerely,

Satomi Iwai

Associate professor

Laboratory of Small Animal Surgery 2

School of Veterinary Medicine

Kitasato University

35-1, Higashi23, Towada-city, Aomori, 034-8628

E-mail: [iwai@vmas.kitasato-u.ac.jp](mailto:iwai@vmas.kitasato-u.ac.jp)

***Response to Reviewer 1:*** I am very grateful for the valuable time and effort you have put into reviewing my paper. I have carefully considered your comments and made the relevant changes in the manuscript.

1. Abstract (page 4, line 45): Add “possibly” to the sentence, because the authors did not unequivocally prove that renal fibrosis was caused by TGF-b1.

**Response:** Thank you for your suggestion. I have added the word. ***(Line 39)***

2. Describe the references precisely. References 5–7 on page 5 used mesenchymal stem cells, and not induced stem cells such as iPS cells. Reference 10 simply shows that NPCs can be induced from patient-derived iPS cells and does not demonstrate the formation of interspecies chimeric nephrons.

**Response:** Thank you for your comment. I have corrected the text. Since the content was misleading to the reader, a description of iPS cells has been added. ***(Lines 66-76)***

3. The authors retracted the figure showing apoptosis, because they were unable to provide a more convincing stained image than that in the first submission, in which the positive staining was hardly distinguishable from the background staining. Nonetheless, the quantitative data based on such unreliable staining are still present in the manuscript and figure. If the authors consider the quantitative data to be reliable, that is fine. Otherwise, Figure 5E and 5F, as well as the related descriptions in the text, should be deleted.

**Response:** We agree with your assessment. We believe that the data are reliable because we had the images checked daily by individuals who are experts in checking details of stained images. However, the image was certainly unclear, so I have enlarged the image to make it easier to understand. I would appreciate it if you could check it. ***(Lines 437-442, Figure 5E)***

4. Figure 5A and 5C should be presented in a larger size, so that readers can recognize the abnormal histology in detail.

**Response:** Thank you for your suggestion. Figures 5 A and C were corrected by enlarging the images.

5. Although the initial sample numbers are described for the schematic in Figure 1, the relevant sample numbers should also be described for all figures and tables that contain statistical analyses. For example, Table 3, Figure 3B and 3C, and Figure 5B and 5D.

**Response:** As you pointed out, the number of samples was included in the figures and tables. ***(Table 3, Figures 3B and C and 5B and D)***

***Response to Reviewer 2:*** Thank you very much for spending your valuable time to review my manuscript.

***Response to Reviewer 3:*** Thank you for taking the time to comment on our manuscript again. I have made corrections in consideration of your comments.

**Minor points:**

1) Introduction, line 59. “alternatives treatment” should be “alternative treatment”.

**Response:** Thank you for your keen observation. This has been revised. ***(Line 54)***

2) Introduction, lines 69 to 70. “induced stem cells” should be “induced pluripotent stem cells”.

**Response:** Thank you for your comment. This has been changed because it refers to mesenchymal stem cells. ***(Lines 66-67)***

3) Introduction, line 74. “NPCs with induced pluripotent stem cells” should be “NPCs differentiated from induced pluripotent stem cells”.

**Response:** We agree with your assessment. This has been revised in ***Line 72-73.***

4) Materials and method, lines 144 to 147. While it is described that the first group had left recipient kidney removed, Fig. 1A indicates that right recipient kidney is removed and used for SWPU.

**Response:** Thank you for providing these insights. The entire Figure 1 was confirmed again and the statement about SWPU has been revised.

5) Materials and method, lines 150 to 152. While it is described that the MNB2 is located to the head side of MNB1, Fig. 1A indicates that the opposite positions of MNB1 and MNB2.

**Response:** We agree with you and have incorporated this suggestion throughout the Figure.

6) Materials and method, lines 235 to 238. The two sentences seem grammatically incorrect and should be revised.

**Response:** I have rechecked the sentences; the entire manuscript has been edited by a native English speaker ***(Lines 238 - 242)***

7) Materials and methods, line 238. “Day28” should be replaced by “Day56”.

**Response:** Thank you for your careful reading. I have revised it. ***(Line 241)***

8) Results, lines 382 to 394. Fig. 3A should be cited in the text.

**Response:** Thank you for your comment. Fig 3A has been cited. ***(Line 392)***

9) Results, line 396. “Histopathological” should be “histopathological”.

**Response:** I have made the revision. ***(Line 401)***

10) Results, line 455. “Above figure” should be “Upper figure”.

**Response:** Thank you for your suggestion. I have made the revision. ***(Line 461)***
