# Supplementary material for: Timing urinary tract reconstruction in rats to avoid hydronephrosis and fibrosis in the transplanted fetal metanephros as assessed using imaging
Source: PLoS One. 2021 Jan 15;16(1):e0231233. doi: 10.1371/journal.pone.0231233 (PMC7810319; doi:10.1371/journal.pone.0231233)
Supplement: Attachment — Submitted filename: Response_to_Reviewers.docx [file pone.0231233.s005.docx]

December 15, 2020

Dr. Franziska Theilig

Academic Editor

*PLOS ONE*

Dear Editor:

Thank you again for inviting us to submit a revised draft of our manuscript titled, “Timing urinary tract reconstruction in rats to avoid hydronephrosis and fibrosis in the transplanted fetal metanephros as assessed using imaging.” The initial draft was submitted to *PLOS ONE* on March 17, 2020, with manuscript ID number PONE-D-20-07683.

We appreciate the time and effort you and the reviewers have dedicated in providing insightful feedback on ways to strengthen our paper. We believe that our edits, highlighted in the manuscript in red, and the responses to reviewer comments provided below have satisfactorily addressed all the issues noted.

Thank you for your consideration. I look forward to hearing from you.

Sincerely,

Satomi Iwai

Associate professor

Laboratory of Small Animal Surgery 2

School of Veterinary Medicine

Kitasato University

35-1, Higashi23, Towada-city, Aomori, 034-8628

E-mail: [iwai@vmas.kitasato-u.ac.jp](mailto:iwai@vmas.kitasato-u.ac.jp)

***Response to Reviewer 1:*** Thank you for your valuable comments. We learned a lot from your comment. Please check our revised version.

**Minor corrections:**

1. Abstract (Line 39 on page 4): thereby decreasing the fibrosis caused possibly by transforming growth factor-b1.

**Response:** Thank you for your suggestion. We have revised the sentence. ***(Line 40)***

2. Line 442 on page 29: Fig 5G (not Figs 5G).

**Response:** Thank you for your careful review. We have corrected the word. ***(Lines 444-445)***

***Response to Reviewer 2:*** Thank you for taking time to review our manuscript.

***Response to Reviewer 3:*** Thank you for taking the time to review our manuscript again. We have made corrections per your comments. For English, we asked for native proofreading again. Thank you for your confirmation.

**Minor points:**

1) Discussion, lines 528-529. “the transplanted metanephros regenerates recipient-derived blood vessels and is chimerized with the donor’s metanephros”. The authors should confirm whether this sentence is grammatically correct. I think “the transplanted metanephros regenerates recipient-derived blood vessels and is chimerized with the vessels” is correct.

**Response:** Thank you for your comment. This has been revised as per your comment. ***(Line 530–532)***

2) Figure 5E. TUNEL(+) cells are more frequently observed in SP group than in 28 UR group, which is opposite to the results of Figure 5F. The authors should replace Figure 5E with more representative images.

**Response:** Thank you for your comment. In Fig 5E, the results of the SP and 28UR groups were reversed. We have corrected this. We apologize for this oversight on our part.
